# Successful ageing in older persons belonging to the Aymara native community: exploring the protective role of psychosocial resources

**DOI:** 10.1080/21642850.2019.1691558

**Published:** 2019-11-18

**Authors:** Lorena P. Gallardo-Peralta, Esteban Sánchez-Moreno

**Affiliations:** a Faculty of Social Work, Universidad Complutense, Madrid, Spain; b School of Social Work, University of Tarapacá, Arica, Chile; c Research Institute for Development and Cooperation (IUDC), Universidad Complutense, Madrid, Spain

**Keywords:** Successful ageing, indigenous Andeans or Aymara, health, community and psychosocial resources

## Abstract

**Objectives:** The aim of this study is to analyse the process of successful ageing in older persons who state their belonging to a native Chilean ethnic group. There has recently been a notable increase in interest regarding analysis of the cultural processes and variables associated with successful ageing. However, there is a lack of studies analysing successful ageing in native ethnic groups; that is, ethnic communities living in their natural environmental surroundings.

**Methods:** A cross-sectional research design was used. The sample was composed by 232 indigenous Aymara persons aged over 60 years who live in the far north of Chile. Data were collected using a questionnaire made up of validated measurement scales for successful ageing, community support, quality of life, religiousness/spirituality, and health.

**Results:** The results suggest that indigenous persons age successfully, particularly in the context of physical functionality. Successful ageing is positively related with community integration, social support from informal systems (social groups), quality of life, and religiousness (forgiveness). In contrast, successful ageing is negatively related with depression.

**Conclusions:** Cultural practices and an active lifestyle are ethnic cultural resources enabling persons to successfully cope with ageing. The results suggest that interventions would benefit from incorporating actions within a context of community integration. Particular attention to preventing deterioration in mental health would help to foster a successful ageing process.

## Background: successful ageing and ethnicity

The concept of successful ageing (Rowe & Kahn, 1987) refers to a paradigm that examines the ageing process in terms of optimal physical, cognitive, psychological and social functioning. It avoids characterising old age as a stage of decline or of progressive and irreversible loss of capacity. The ageing process effectively implies an accumulation of experiences, some of them negative, including disease, loss of independence and the weakening of social networks. However, some older persons are capable of adapting to this new scenario by using their psychosocial resources; they ultimately manage to age successfully (Jopp & Rott, 2006). Older persons may, therefore, participate in activities to improve, change or transform their lifestyles, and hence improve their chances of better ageing (Cheng, 2014). In this vein, the current definition of successful ageing focuses mainly on the process of adaptation by older persons to the changes inherent to old age and the empirical evidence suggests that the key lies in the capacity to successfully adapt to the new circumstances of old age, which would give rise to the perception that one is ageing well (Cho, Martin, & Poon, 2015; Kleineidam et al., 2018). The present piece of research focuses on the elements relevant for the understanding of successful ageing in a sample made up of indigenous Aymara older adults living in the north of Chile, with two aims. First, to reduce the scarcity of studies focused on the analysis of successful ageing in the case of native indigenous groups. Second, to increase available significative knowledge for the design of interventions with those ethnic groups from the intercultural perspective.

Previous research identifies specific groups of variables that are explanatory or predictive of successful ageing, such as age, income, educational level, presence of medical conditions, and hearing difficulties, among others (Fernández-Ballesteros et al., 2010; Kim & Park, 2017). Of all of these, the process of health (both physical and mental) has received the most attention in previous research (Cho et al., 2015; Hilton, Gonzalez, Saleh, Maitoza, & Anngela-Cole, 2012; Jopp et al., 2014; Parslow, Lewis, & Nay, 2011), given its strong association with successful ageing. In any case, the analytical perspective of this study leads toward an emphasis on those predictor variables that are linked to processes with particular significance in social research and intervention. Of note in this regard is the potential importance for successful ageing of variables such as community social support, quality of life, religiousness/spirituality, and physical and mental health. Various studies confirm the intense and significant relationship between social support networks and successful ageing (Hilton et al., 2012; Parslow et al., 2011; Pruchno, Wilson-Genderson, Rose, & Cartwright, 2010). It is important to note that integration in community social networks can improve the physical and mental health of older persons (Jopp et al., 2014). Additionally, the empirical evidence suggests that quality of life is positively related to successful ageing (Li et al., 2014). Moreover, numerous studies stress the close and positive relationship between religiousness/spirituality and successful ageing (Hilton et al., 2012; Pruchno et al., 2010), taking into account that both are personal resources principally linked to psychological and mental well-being (Dahany et al., 2014).

In the case of quality of life (hereinafter, QoL) during ageing, studies conducted in Chile show that the variables or dimensions related with QoL among older Chilean persons are having social support networks, enjoying good health (physical functionality and self-efficacy), having the capacity for self-acceptance and belonging to a protected physical environment. In this regard, the role played by social support networks (specifically relationships with family, friends and the community) is recurrent in the evaluation of QoL of older Chilean persons. Health, frequently in the form of capacity for self-efficacy or independence (functional capacity), is also of particular importance, with an emphasis on the positive influence of physical activity for this dimension. Along these lines, adults experience various ailments and illnesses, but ‘adaptive capacity’ is key, in terms of new conditions or even limitations on dealing with the problems experienced in everyday life. Finally, belonging to a protected physical environment is positively related with QoL, with an emphasis on the perception of housing and/or the neighbourhood as spaces that are removed from problems such as violence, drug addiction and alcoholism (Herrera, Barros, & Fernández, 2011; Osorio, Torrejón, & Anigstein, 2011; Torres, Quezada, Rioseco, & Ducci, 2008; Urzúa, Bravo, Ogalde, & Vargas, 2011). The impact of spirituality on QoL among older Chileans has also been confirmed, with it having ‘a significant role for spiritual experiences in the ability of elderly people to find equilibrium in terms of the implications of the ageing process in the physical, psychological, social and environmental spheres’ (Gallardo-Peralta, 2017, p. 11).

Within this general research framework, there has recently been a notable increase in interest regarding analysis of the cultural context as a factor associated with successful ageing (Lewis, 2011; Pace & Grenier, 2017; Torres, 2015). There is an affirmation of the importance to gerontology of developing theoretical models that are sensitive to cultural diversity, which would contribute toward improved design and implementation of interventions. Studies have been carried out along these lines with ethnic minorities in the United States and in some European countries (Bowling, 2009; Cené et al., 2016). However, there is a lack of studies analysing successful ageing in native ethnic groups; that is, ethnic communities living in their natural environmental surroundings who have become ethnic minorities within their own geographical and territorial context.

Research does exist that attempts to analyse specific aspects of the ageing process in ethnic communities with such characteristics, above all in the Latin American context, given the importance of native ethnic groups in its social and cultural environment. This is the case for studies concerning native Andean communities, within which context research has focused on indigenous older persons and various measures of well-being. The results of these studies show potential situations of social risk: dependency on the areas of mental functions and communication; worsening perception of quality of life related with health; prevalence of depressive symptoms, mainly in women (Gallardo-Peralta, Sánchez-Moreno, Barrón, & Arias, 2015; Mella, Alvear, Carrillo, & Caire, 2003). However, the evidence only offers indirect indicators with regard to the ageing process, not entailing a specific and detailed analysis of the process of successful ageing among indigenous older persons in the case of native ethnicities.

This research is intended to undertake such an analysis in the case of the indigenous Andean Aymara community. The Aymara people are descended from the *Tiwanaku* culture and hence continue to maintain frequent family and community exchanges with Bolivia and Peru. In Chile, the Aymara live in the far north of the country and their natural surroundings are the highland and valley areas of the foothills of the Andes. Their social organisation is based on the *ayllu* community model and is highly family focused. The villages and dwellings that they inhabit experience significant problems in terms of supply of services such as electricity, drinking water, sewage treatment and communication and connection with the rest of Chile, which has in recent decades led to the depopulation and ageing of native communities. The main labour activities of the indigenous Aymara can be distinguished according to the geographical location of the settlement. Those living in the highlands carry out livestock work, such as herding (llamas, alpacas, guanacos). Those residents in the foothills mainly cultivate garlic and oregano. In the geographical areas closer to the sea, the norm is agricultural work such as the cultivation, irrigation and harvesting of fruit in particular (Carrasco & González, 2014). It is important to clarify that the rural Aymara economy is predominantly agricultural and it may even be affirmed that, in the far north of Chile, this rural agricultural economy is basically controlled by the Aymara community from production to delivery of products to consumers (Gundermann, González, & Durston, 2014).

Interactions between older adults and members of their close and extended family tend to occur at religious festivals or other Aymara rites that are still celebrated in the villages, in which space older people play a leading role in cultural transmission. The significance of the role of older persons in maintaining indigenous cultural practices is shown in their participation in the social tasks (attendance at community meetings, maintaining the native language, and so on) that represent an essential part of the routine of highland families (Gallardo-Peralta, Sánchez-Moreno, & Rodríguez-Rodríguez, 2019; Gavilán, 2002). It is important to note that an intense process of assimilating indigenous peoples (known as *chilenización*) took place from the final decade of the nineteenth century, which contributed to a significant erosion of certain features of social interaction in the indigenous cultural context (language, medical practices, religious practices, etc.). In fact, the Aymara use Spanish as their working language, reserving the use of the Aymara language for private or indigenous community spaces.

This work seeks to contribute to reducing the gap in the empirical evidence that is available regarding SA in the case of indigenous communities. Our study examines successful ageing among older persons of Aymara ethnicity living in the north of Chile, in order to understand the characteristics of the process of successful ageing among older persons belonging to native ethnicities. Our study is intended to analyse the relationship between psychosocial processes (community social support, quality of life, religiousness/spirituality and health) and successful ageing among indigenous older persons.

## Method

### Participants and procedure

The study was quantitative and cross-sectional, involving the participation of 232 indigenous older persons living in the far north of Chile. The design of the sample was non-probabilistic, given the difficulties found for it working with the indigenous population under study. In order to obtain an appropriate level of structural representativeness, a quota sample by sex, age, ethnicity and place of residence was designed. The fundamental aims were ensuring (1) that the sample reflected the characteristics of the population and (2) that the sample was sufficient to carry out the statistical analysis. Along these lines, the participants characteristics described in the results section adequately reflect the main social features of the population. The inclusion criteria were being aged 60 or over, belonging to the indigenous Aymara ethnicity and not suffering from serious cognitive deterioration. Some members of the research team (especially social workers) had enjoyed previous access to some communities from which participants were recruited, which enabled the technical team to obtain access without difficulty. These agents carried out an initial selection of participants based on the inclusion criteria. The experience and knowledge of the community of social workers contributed to a recruitment process that enabled the identification of persons with dementia (excluded from eligibility). Only participants without missing information on any of the variables considered were included in the study. The questionnaire was applied via personal (face-to-face) interview, having first obtained the informed consent of participants.

The main source of bias in the present study was the administration of the questionnaire and the understanding of the measurement scales by the participants. It was essential to ensure that all members of the sample clearly and consistently understood the content of the different measurement instruments. To that end, a pilot measure was taken from a group of 33 people, none of which were part of the final sample. The pilot study allowed the questionnaire to be refined such that the interviewers were provided with clear instructions and definitions for the application of the measurement instruments. Qualified professionals administered it between June and August 2015. To reduce the interviewer variability effect on information gathering, all interviewers underwent a training programme in order to ensure that the interviewers had the skills and knowledge to correctly carry out interviews. This involved specific training on the research questionnaire. Similarly, particular attention was paid to the design of the questionnaire with the aim of reducing misinterpretation of information by interviewers.

The Ethics Committee of the University of Tarapacá and the National Council for Science and Technology of Chile approved and monitored the ethical aspects of the study. All procedures performed in studies involving human participants were in accordance with the ethical standards of the institutional research committee and with the 1964 Helsinki declaration and its later amendments or comparable ethical standards.

### Measures

Seven measurement scales were used to adequately measure the psychosocial dimensions included as part of the study aim. All data were self-reported.

#### Successful ageing

The Successful Ageing Inventory (SAI) devised by Troutman, Nies, Small, and Bates (2011) was used. This is made up of 20 items, a Likert scale is used, ranging from (0) totally disagree to (4) totally agree. The values for the different dimensions are added together and a score is obtained that varies from 0 to 80. Higher scores indicate successful ageing. In specific terms, scores from 0 to 25 indicate unsuccessful ageing, scores from 26 to 53 suggest moderately successful ageing, and scores from 54 to 80 indicate successful ageing. This inventory has been validated in Chilean, indigenous and non-indigenous elderly people, proving to be a reliable and adequate instrument for this population (Gallardo-Peralta, Cuadra-Peralta, Cámara-Rojo, Gaspar-Delpino, & Sánchez-Lillo, 2017). Cronbach’s alpha was .92.

#### Community support

The Perceived Social Community Support Questionnaire devised by Gracia, Herrero, and Musitu (2002) was applied. This questionnaire has a total of 25 items distributed across four dimensions and each dimension is made up of a sub-scale that can be separately analysed: community integration; community participation; social support in informal systems and social support in formal systems. The categories for responding to all of these questions follow a Likert-style design where (1) means strongly disagree and (5) means strongly agree. Scores are obtained for each dimension evaluated. This questionnaire has been validated in the Chilean population, including indigenous elderly people (Gallardo-Peralta & Gálvez-Nieto, 2018). Cronbach’s alpha was .63.

#### Quality of life

The WHOQOL-BREF scale, from the quality of life group of the World Health Organization (1998), was used. It is made up of a total of 26 items. This questionnaire offers a partial score for each dimension and a general/overall one. A higher score means a better quality of life. This questionnaire is widely used in Chilean elderly people and has a validation in this age group (Espinoza, Osorio, Torrejón, Lucas-Carrasco, & Bunout, 2011). Cronbach’s alpha was .89.

#### Religiousness/spirituality

The Brief Multidimensional Measure of Religiousness/Spirituality (BMMRS) from the Fetzer Institute (1999) was applied. This questionnaire evaluates different theoretical aspects of religiousness and spirituality. It has a total of 38 questions, which are aggregated into the following dimensions: daily spiritual experiences; values/beliefs; forgiveness; religious practices; religious/spiritual coping; religious support; religious/spiritual history; commitment; organisational religiousness; religious preference; and general self-classification regarding religiousness and spirituality. This questionnaire has been validated in the Chilean population, confirming its bifactorial structure: spirituality and religiosity (Gallardo-Peralta, Cuadra-Peralta, & Veloso-Besio, 2018). Cronbach’s alpha was .88.

This study evaluated health in terms of mental health (depression), main health problems (most recurrent illnesses) and dependency in daily activities. The following instruments were applied to assess health in these contexts:

#### Mental health

The existence of depressive symptoms was evaluated via the Geriatric Depression Scale (GDS) developed by Brink et al. (1982). The original scale consisted of 30 items, but the abbreviated version (containing 15 items) was used. This version maintains the effectiveness of the original scale, improving ease of administration. The instrument records the presence of 15 symptoms of depression, with a resulting score from 0 to 15. A score of ≥5 indicates depression, with the following ranges indicative of severity: 5–9 points for mild, 10–15 for moderate-to-severe depression. It is a scale widely used to evaluate depression among older Chilean persons (Hoyl, Valenzuela, & Marín, 2000). Cronbach’s alpha was .81.

#### Main health problems

The Health Problems Questionnaire produced by Herrera, Barros, and Fernández (2007) was used. This instrument was specifically developed to measure the most recurrent illnesses in the population of older persons in Chile, within the framework of the National Survey of Quality of Life in Old Age. It constitutes an inventory/checklist made up of 14 pathologies.

#### Dependency

The Barthel Activities of Daily Living (ADL) Index, developed by Mahoney and Barthel (1965), was applied. Each activity is assigned a score (5, 10, 15) based on the time spent carrying it out and the need for assistance in doing so, resulting in a final score from 0 to 100. The scores obtained on the scale indicate the following: 0–20: total dependence; 21–60: severe dependence; 61–90: moderate dependence; 91–99: little dependence; and 100: independence. The Barthel ADL Index is applied in Family Health Centres in Chile. Cronbach’s alpha was .90.

### Data analysis

Two stages of data analysis were implemented in order to address the aims of the study. First, the descriptive statistics corresponding to the scores for the successful ageing questionnaire and the other study variables. Second, a hierarchical regression analysis was carried out for the ‘successful ageing among indigenous older persons’ variable, which entailed the creation of four models. Included in all of these were the following control variables: sex (0 = male, 1 = female), age and marital status (0 = single, 1 = with partner). In model 1, the dimensions of community social support (integration in community/neighbourhood, participation in community/neighbourhood, informal social support and formal social support) were included. Model 2 integrated the quality of life variable. Model 3 added together the scores for the various dimensions of religiousness/spirituality (daily spiritual experiences, values/beliefs, forgiveness, private religious practices, religious/spiritual coping and religious support). Finally, model 4 included mental and physical health (depression, health problems and dependency). Version 23 of the IBM-SPSS programme was used to carry out the analyses.

## Results

### Participants characteristics

The main socioeconomic characteristics were: 65% women and 35% men. In terms of age, 58% were between 60 and 69 years old, 32% between 70 and 79, and 10% over 80 years old (mean = 69.54 years; SD = 7.38). With respect to marital status, 47% had a partner (whether married or cohabiting), 23% were widows/widowers, 17% divorced or separated, and 13% single. Concerning the level of education, 24% had not attended school, 38% had completed primary education, 30% secondary education, and 8% had completed higher education. In terms of place of residence, 66% lived in an urban area (Arica), 23% in highland areas (Putre, Belén, Visviri), and 11% in foothills (Codpa and its surrounding area). Concerning financial background, 66% were still working for financial remuneration and 55% contributed the highest amount to their household (head of household).

### Descriptive statistics

Table 1 shows the descriptive statistics and Pearson correlation values for the study variables. The results suggest that the sample under study would be categorised as ageing successfully, especially when functional performance is considered. On the other hand, intrapsychic factors and gerotrascendence result in lower relative levels when compared with the rest of dimensions of SAI.

**Table 1.** Descriptive statistics and correlation coefficients (Pearson) for study variables.

Domains of successful ageing	Mean	SD	Range														
Functional performance mechanisms	7.20	1.54	0–8														
Intrapsychic factors	23.81	4.92	0–28														
Gerotranscendence	19.83	3.94	0–24														
Spiritual dimensions	6.14	2.03	0–8														
Purposefulness/life satisfaction	10.72	2.27	0–12														
Correlations Coefficients				2	3	4	5	6	7	8	9	10	11	12	13	14	15
1. Successful Ageing (global)	67.7	12.16	0–80	.17**	.08	.22*	.09	.32**	.13*	.14*	.25**	.13*	.10	.02	−.36**	−.07	−.06
2. Community integration	17.92	3.58	5–25	–	.20**	.11	.28**	.18**	.15*	.07	.09	.06	.16*	.11	−.17**	−.08	−.11
3. Community participation	17.48	2.35	6–30		–	−.05	.07	.02	−.02	.01	.08	.19**	.04	.25**	−.06	.02	−.06
4. SS from informal systems	42.63	5.64	10–50			–	.18*	.33**	.37**	.19*	.10	.11	.36**	.20*	−.10	−.01	−.13
5. SS from formal systems	14.05	3.46	4–20				–	.03	−.07	.02	.03	−.02	.07	.08	−.06	−.07	.01
6. Quality of life	35.87	3.50	26–44					–	.25**	.19**	.08	.12	.14*	.00	−.47**	−.29**	−.24**
7. Daily spiritual experiences	12.45	6.82	6–36						–	−.51**	−.40**	−.49**	−.47**	−.29**	.12	.09	.04
8. Values/Beliefs	2.86	1.07	2–8							–	−.37**	−.33**	−.34**	−.23**	.06	.04	.08
9. Forgiveness	4.04	1.52	3–12								–	−.44**	−.31**	−.17**	.01	.01	.10
10. Private religious practices	17.78	6.67	5–25									–	−.39**	−.40**	.04	.11	.10
11. Religious/spiritual coping	14.75	3.45	6–28										–	−.29**	.00	.13*	.00
12.Religious/congregational support	12.96	2.97	4–15											–	.02	.02	.09
13.Depression	3.17	2.78	0 –15												–	.10	−.18**
14.Health problems	1.54	1.37	0–14													–	−.08
15.Dependency	97.26	9.39	0–100														–

SD = standard deviation; SS = Social support; *p < .05. **p < .01.

### Main results

Table 2 shows the results obtained in the hierarchical regression analysis for the ‘successful ageing’ variable. Though the four models are statistically significant, in specific terms models 3 and 4 entail a significant increase in their capacity to explain successful ageing, with notable explained variance percentages (27.3% in model 3 and 33.6% in model 4). The inclusion of the dimensions of religiousness/spirituality and depression thus makes a significant contribution to understanding successful ageing among older indigenous Chilean persons. When undertaking a specific analysis using the contrasted models, the main predictive variables are observed to be the following: for model 1 (community social support), age (*β* = −.252; *p* < 0.01) and community integration are the main predictive variables (*β* = 0.22; *p* < 0.01) along with social support from informal systems (*β* = 0.20; *p* < 0.05). In model 2 (quality of life), age again remains (*β* = −0.20; *p* < 0.05) as does community integration (*β* = 0.19; *p* < 0.05), and quality of life is added (*β* = 0.21; *p* < 0.05). Model 3 (religiousness/spirituality) retains age (*β* = −0.19; *p* < 0.05), community integration (*β* = 0.21; *p* < 0.05), social support from informal systems (*β* = 0.21; *p* < 0.05), and quality of life (*β* = 0.19; *p* < 0.05), with forgiveness being added (*β* = 0.25; *p* < 0.01). Finally, in model 4 (mental and physical health), social support from informal systems (*β* = 0.20; *p* < 0.05) and forgiveness (*β* = 0.27; *p* < 0.01) remain, with depression added (*β* = −0.28; *p* < 0.01). In sum, successful ageing is positively related with community integration, social support from informal systems, quality of life and forgiveness. In contrast, successful ageing is negatively related with age and depression.

**Table 2.** Hierarchical regression predicting successful Ageing, from social and psychological variables.

	MODEL 1 COMMUNITY SOCIAL SUPPORT			MODEL 2 QUALITY OF LIFE			MODEL 3 RELIGIOUSNESS/SPIRITUALITY			MODEL 4 MENTAL AND PHYSICAL HEALTH		
	B (95% CI)	S.E.	***β***	B (95% CI)	S.E.	***β***	B (95% CI)	S.E.	***β***	B (95% CI)	S.E.	***β***
*Sociodemographic control*												
Sex	−1.00 (−5.93, 3.92)	2.49	−0.03	−0.72 (−5.57, 4.11)	2.44	−0.02	−1.00 (−6.07, 4.05)	2.55	−0.03	−0.12 (−5.09, 4.83)	2.50	−0.00
Age	−0.42 (−0.73, −0.12)	0.15	−0.25**	−0.34 (−0.65,−0.03)	0.15	−0.20*	−0.32 (−0.63, −0.00)	0.16	−0.19*	−0.26 (−0.59, 0.07)	0.16	−0.15
Marital status	−3.82 (−8.56, 0.90)	2.38	−0.14	−3.74 (−8.38, 0.90)	2.34	−0.14	−1.98 (−6.66, 2.69)	2.36	−0.07	−2.29 (−6.85, 2.27)	2.29	−0.08
*Social and psychosocial resources*												
Community integration	0.79 (0.14, 1.44)	0.32	0.22**	0.68 (0.04, 1.32)	0.32	0.19*	0.76 (0.10, 1.42)	0.33	0.21*	0.62 (−0.02, 1.27)	0.32	0.17
Community participation	0.51 (−0.52, 1.54)	0.52	0.09	0.45 (−0.55, 1.47)	0.51	0.08	0.39 (−0.67, 1.45)	0.53	0.06	0.16 (−0.88, 1.21)	0.52	0.02
Support from informal systems	0.49 (0.07–.091)	0.21	0.20*	0.33 (−0.10, 0.76)	0.21	0.14	0.51 (0.04, 0.98)	0.23	0.21*	0.47 (0.00, 0.94)	0.23	0.20*
Support from formal systems	−0.28 (−0.94, 0.36)	0.33	−0.08	−0.32 (−0.96, 0.32)	0.32	−0.09	−0.21 (−0.88, 0.46)	0.34	−0.06	−0.23 (−0.89, 0.41)	0.33	−0.06
Quality of life				0.78 (0.10, 1.46)	0.34	0.21*	0.69 (0.00, 1.38)	0.34	0.19*	0.33 (−0.47, 1.14)	0.40	0.09
*Reliogiosity/Spirituality*												
Daily Spiritual Experiences							−0.23 (−0.30, 0.76)	0.26	−0.11	−0.29 (−0.23, 081)	0.26	−0.13
Values/Beliefs							0.20 (−2.91, 2.50)	1.36	0.01	0.55 (−3.26, 2.15)	1.36	0.04
Forgiveness							2.38 (−4.11, −0.65)	0.87	0.25**	2.55 (−4.23, −0.86)	0.84	0.27**
Private Religious Practices							0.14 (−0.61, 0.32)	0.23	0.06	0.08 (−0.55, 0.37)	0.23	0.03
Religious/Spiritual Coping							−0.51 (−0.23, 1.27)	0.38	−0.14	−0.31 (−0.46, 1.09)	0.39	−0.08
Religious Support							−0.57 (−0.21, 1.53)	0.39	−0.13	−0.52 (−0.24, 1.29)	0.38	−0.12
*Health*												
Depression										−1.28 (−2.13, −0.43)	0.42	−0.28**
Health problems										0.40 (−1.47, 2.27)	0.94	0.04
Dependency										−0.00 (−0.29, 0.27)	0.14	−0.00
*R*^2^	0.15	0.18	0.27	0.33								
*F* change	3.39**	5.28**	2.06***	3.26***								

*p < .05, **p < .01, ***p < .001; S.E.: Standard error.

## Discussion

The results obtained suggest that a process of successful ageing characterises older Aymara persons who participated therein. In this regard, the average score (67.7) was located in the successful ageing interval according to the evaluation criteria of the questionnaire used (scores from 54 to 80 indicate successful ageing). Though this statement is applicable to the various dimensions used of the SAI, the average scores for the ‘functional performance’ (7.20 with a range of 0–8) and ‘life purpose/satisfaction’ (10.72 with range 0–12) categories are of particular note. In general terms, and particularly in these two cases, it is possible to link successful ageing with various cultural elements characteristic of this native ethnicity, which could be positively influencing both the definition and the successful unfolding of ageing.

The older Aymara people have an active lifestyle, to a large extent due to the Aymara organisation of productive and reproductive activities. This circumstance – maintaining labour activity and the specific features of that activity – may contribute to explaining the positive results found for the dimension of ‘physical functionality’ in the SAI. Insofar as older Aymara persons integrate and participate in maintaining indigenous cultural practices (Gallardo-Peralta et al., 2019; Gavilán, 2002), it is to be expected that cultural context will facilitate successful ageing. The results obtained in the regression analysis undertaken as part of the study support this interpretation. These results show that community integration and support from informal systems are positively related with successful ageing. Both factors show a significant association with successful ageing, independently of the role played by quality of life and psychosocial variables such as religiousness, which suggests that the role played by the social, cultural and economic organisation of the Aymara as regards the community is a notably important one (Gavilán, 2002). When we speak of community in the Aymara context, we are particularly referring to an environment entailing what are usually intra-ethnic relationships and social networks. In this context, community is constructed based on the participation of individuals assessed depending on their belonging to a family, which may be direct or extended (Gundermann et al., 2014).

Within this framework, our results reaffirm the importance of quality of life in constituting a key variable in understanding the process of successful ageing, as stated in previous literature (Abdala, Kimura, Duarte, Lebrão, & Santos, 2015; Kleineidam et al., 2018). Moreover, our results confirm the importance of religiousness and spirituality in the successful ageing process among older Aymara persons (Wright, 2015). Of the various aspects of religiousness/spirituality considered in this study, the dimension of ‘forgiveness’ plays a significant role in our analyses. Forgiveness implies the development of cognitive and emotional capacity to empathise with weaknesses, mistakes and defects (whether one’s own, someone else’s, or those of a Higher Being), thus enabling one to forgive oneself, forgive others, and feel forgiven by God (Idler et al., 2003), a characteristic that has been linked to well-being in old age (Cowlishaw, Niele, Teshuva, Browning, & Kendig, 2013). Forgiveness ultimately represents a flexible approach that entails attempting to place oneself in another’s position. This flexibility and ability to adapt to changes in context is one of the characteristic elements of the Aymara culture and community, as previous studies have shown (Carrasco & González, 2014). This ability to adjust to changing circumstances is a cultural characteristic that develops in the area of spirituality and represents a particularly significant element in the process of successful ageing, central to which is satisfactory adaptation to the changes resulting from ageing. Moreover, this plasticity and ability to adapt arise in a cultural context within which – as we have seen – active and on-going participation of older persons in the reproduction/production of the domestic unit and the community are key (Yampara, 1992).

As regards the elements that are negatively related with successful ageing, it is notable that of the three health indicators (dependency, health problems and depression), only deterioration in mental health showed a significant association. The Geriatric Depression Scale, which considers symptoms such as loneliness, lack of meaning in life, abandonment of interests, feelings of boredom, experiencing fear, and feeling useless, was used to measure depressive feelings in our study. On this point it is necessary to emphasise that indigenous Aymara people, especially those living in highland zones, have witnessed the depopulation of their regions. Likewise, they have seen their key social networks affected by the migration of younger people to urban areas (Caroselli, 2013; Wright, 2015). This reduction in family networks can give rise to a strong feeling of loneliness. Other environmental factors that may endanger this group’s mental health include geographical isolation (which may be the result of relative geographical marginality) and the poverty and material deprivation suffered by rural Andean zones (Gundermann et al., 2014). In this social and structural context, our results suggest that the main threat to the successful ageing of older Aymara persons in the sphere of health comes from deterioration in mental health, and specifically the appearance of symptoms of depression (see Gallardo-Peralta et al., 2015).

This study presents certain limitations that must be considered. First, the study is cross-sectional in design, which impedes establishing causal relations among variables. Second, the data analyses performed include a relatively high number of variables measured via scales made up of various items. Although the sample size is adequate, it would be appropriate to take this circumstance into account in evaluating the results and conclusions offered. Third, though the sample adequately represents the composition of the indigenous (Aymara) population in the region of Arica and Parinacota, the results cannot be generalised for the entire indigenous population of Chile. Fourth, the average score for the dependence measure (Barthel ADL index) suggests the possible existence of a ceiling effect in the data analysis, and specifically in the estimation of its association with the outcome variable. It is possible that the measure used is not entirely appropriate for research using a community-based population. Fifth, empirical evidence is not available with regard to the development of successful ageing in the case of indigenous older persons belonging to *native* ethnicities. Therefore, it is not possible to contrast the results obtained in this research with previous data. In this regard, the ground-breaking nature of our study reveals the need for a more profound understanding of the ethnic and cultural determinants of successful ageing. In particular, the study of the ageing process in native ethnicities offers notable potential for the generation of important knowledge concerning the impact of sociocultural processes on the ageing process. In this respect, research undertaken from a qualitative methodological perspective would be particularly significant, as it would permit an analysis of the dimensions of ‘ageing successfully or ageing well’ based on their interpretation in the account of indigenous older persons.

In the contexts of these limitations, present peace of research identify deterioration in mental health (specifically, symptoms of depression) as one of the main threats to successful adaptation to ageing. It is worth emphasising that in terms of health, the role of this variable stands out from others such as physical health in the case of the Aymara people who participated in our study. Previous studies suggest that the psychological deterioration of older persons belonging to native ethnicities is strongly related with situations of social isolation (Gallardo-Peralta et al., 2015). Given the significance that symptoms of depression appear to have in the ageing process, it is necessary to integrate mechanisms into our actions aimed at fostering successful ageing in order to combat feelings of ‘loneliness’ in indigenous older persons.

Our results suggest the existence of indicators of successful ageing for indigenous older persons from the north of Chile. Certain Aymara cultural characteristics appear to define and foster a process of successful adaptation to old age. Indigenous persons aged over 60 years have constructed their individual, family and group identity based on the notion of *being Aymara* (Gundermann et al., 2014), constructing an ethnic, cultural and symbolic space concerning the native cultural core or essence that powerfully influences the organisation of their lives. Our results suggest that it is essential to take this context into account in order to understand the life stories of older persons in their (successful) process of ageing.

As a consequence, this research opens up a space for reflection in the field of intercultural interventions.

More specifically, our results support the usefulness of and need for an ethnically sensitive approach based on an anti-racist, anti-oppressive and anti-discriminatory perspective of social intervention. This is rooted in the idea that a recognition of the cultural values, cultural needs and differences of local ethnic groups includes a practice focused on developing positive minority identities, taking affirmative action and empowering indigenous communities (Urh, 2008, pp. 121–122). This approach can guide interventions with indigenous Chilean communities that are seeking to maintain their cultural customs (for example, respecting their family and community values, which differ from the rest of the Chilean population) and this tends to be a recurring theme in spaces designed to increase social participation for indigenous older adults when they interact with professionals. Designing healthcare spaces (such as primary healthcare centres) that incorporate elements of indigenous culture implies the creation of a particularly important community area. With the support of psychosocial intervention professionals, older persons can also design and implement community resources that address various processes related with successful ageing in a comprehensive manner, incorporating essential elements of the indigenous culture. These include health, nutrition, companionship, mobilisation of social support, coping with shared experiences in the ageing process, and community participation. Self-managed community dining spaces and places for participation in decision-making relating to space/urban planning are two examples of this. Along these lines, the present study confirms the protective role of psychosocial resources, and specifically of integration, community social support and religiousness. In this regard, our results suggest that these resources are significant for interventions and fostering positive ageing for ethnic minorities in general, and for indigenous native communities in particular. Moreover, research has identified the Indigenous research paradigm, involving indigenous knowledge, cultures, and protocols in relation to the place and nation within whose territory the research is undertaken (Pidgeon, 2018). In other words, this paradigm entails the relocation of social intervention to produce research that is close to and contextualised by an indigenous cosmovision. From the scope of Latin American social disciplines, specifically in Chile, there is a need to study in depth the impact of indigenous cultural practices that promote well-being in advanced ages (Gallardo-Peralta et al., 2019). This study is a first approach to the promotion of ageing successfully. It is necessary to work on the design of intercultural gerontological intervention sensitive to the world view of indigenous communities, with the aim to promote healthy and natural lifestyles.

The design of psychosocial interventions with an ethnic perspective should take into account the debate revolving around the cultural construction of the SA concept. In this sense, it is worth emphasising that the dimensions defining and comprising this concept are not necessarily equivalent for all ethnic groups and in all cultural contexts. Some of its dimensions may be more relevant in designing the social intervention than others, bearing in mind the specific cultural and ethnic context for which the intervention is designed and constructed (Afshar, Franks, Maynard, & Wray, 2002). The objective is to achieve better clarification of the components of each process for the population (Kleineidam et al., 2018). At the heart of the debate is the need to specify, as far as possible, the dimensions and processes that would be part of the ‘culturally-biased’ definitions of SA. This observation is particularly potent in the case of the ethnic group at hand (the Aymara community) or any other that shares the combination of two elements. The first is the importance of maintaining certain indigenous cultural practices, both specifically in the area of health and generally in social interaction. The second element is the fact that this community competently interacts and participates in the daily life of the broader society (that is, the non-indigenous society), with which it shares customs, social and economic exchanges and the use of the same language (Pelcastre-Villafuerte et al., 2017).

Finally, our results also identify depression as one of the main threats to successful adaptation to ageing. It is worth emphasising that in terms of health, the role of this variable stands out from others such as physical health in the case of the Aymara people who participated in our study. Previous studies suggest that the psychological well-being of older persons belonging to native ethnicities is strongly related with situations of social isolation (Gallardo-Peralta et al., 2015). Given the significance that symptoms of depression appear to have in the ageing process, it is necessary to integrate mechanisms into our actions aimed at fostering successful ageing in order to combat feelings of ‘loneliness’ in indigenous older persons.
